# Proteomics Complementation of the Rat Uterotrophic Assay for Estrogenic Endocrine Disruptors: A Roadmap of Advancing High Resolution Mass Spectrometry-Based Shotgun Survey to Targeted Biomarker Quantifications

**DOI:** 10.3390/ijms22041686

**Published:** 2021-02-08

**Authors:** Laszlo Prokai, Fatima Rahlouni, Khadiza Zaman, Vien Nguyen, Katalin Prokai-Tatrai

**Affiliations:** Department of Pharmacology and Neuroscience, University of North Texas Health Science Center, Fort Worth, TX 76107, USA; Fatima.Rahlouni@my.unthsc.edu (F.R.); Khadiza.Zaman@unthsc.edu (K.Z.); Vien.Nguyen@unthsc.edu (V.N.); Katalin.Prokai@unthsc.edu (K.P.-T.)

**Keywords:** endocrine disruption, rat uterus, estrogen-regulated proteins, 17β-estradiol, liquid chromatography–mass spectrometry, label-free proteomics, high resolution mass spectrometry, protein networks, targeted proteomics, bisphenol A

## Abstract

The widely used rat uterotrophic assay to assess known and potential estrogenic compounds only considers uterine weight gain as endpoint measurement. To complement this method with an advanced technology that reveals molecular targets, we analyzed changes in protein expression using label-free quantitative proteomics by nanoflow liquid chromatography coupled with high-resolution mass spectrometry and tandem mass spectrometry from uterine protein extracts of ovariectomized rats after daily 17β-estradiol exposure for five days in comparison with those of vehicle-treated control animals. Our discovery-driven study revealed 165 uterine proteins significantly regulated by estrogen treatment and mapped by pathway analyses. Estrogen-regulated proteins represented cell death, survival and development, cellular growth and proliferation, and protein synthesis as top molecular and cellular functions, and a network found with the presence of nuclear estrogen receptor(s) as a prominent molecular node confirmed the relevance of our findings to hormone-associated events. An exploratory application of targeted proteomics to bisphenol A as a well-known example of an estrogenic endocrine disruptor is also presented. Overall, the results of this study have demonstrated the power of combining untargeted and targeted quantitative proteomic strategies to identify and verify candidate molecular markers for the evaluation of endocrine-disrupting chemicals to complement a conventional bioassay.

## 1. Introduction

Endocrine-disrupting chemicals (EDCs) are a class of agents that interfere with the biological actions of hormones, and there has been significant public concern about their adverse effect on the environment and on human health [1]. EDCs are broadly categorized according to the hormones that they interfere with estrogens, androgens, or thyroid hormone activities. The US Environmental Protection Agency (EPA) has set up the Endocrine Disruptor Screening Program to test the tens of thousands of chemicals for suspected endocrine disruption based on a directive from section 408(p) of the Federal Food, Drug and Cosmetic Act, the Food Quality Protection Act and the Safe Drinking Water Act of 1996 to develop a chemical screening program using appropriately validated methods to determine whether substances may have hormonal effects [2,3,4]. Agents that mimic the effect of endogenous human estrogens are the most recognized class of EDCs.

For the vast majority of these compounds that are tested for estrogenic effects in the uterus, the “gold standard” in vivo uterotrophic rat assay is utilized, and they may be tested in vitro on rodent, yeast, and human cell lines as part of the complementary battery of assays implemented in the EPA’s two-tier testing program [2,3,4,5,6,7,8,9,10]. The uterotrophic assay uses either sexually immature female rats or adult ovariectomized (OVX) female rats, where there is no significant source of endogenous estrogens. In either version of the assay, multiple doses of a test compound are administered over consecutive days (a minimum of three days either orally or subcutaneously). Agents that do have estrogenic effects cause uterotrophic response due to imbibition of water and growth of the uterine cells. Statistically significant uterine weight increases compared to controls give a positive result.

The current in vitro and in vivo methods are not without limitations. The cell lines are not properly able to recapitulate the in vivo environment of the uterus within the body. On the other hand, the rat uterotrophic assay merely considers the uterine weight gain as an endpoint of estrogenicity without taking into account all factors that play a role in exerting an estrogenic effect on the organ and body [9,10,11,12,13,14,15]. The need for advanced testing to evaluate the safety of tens of thousands of xenobiotics in the environment has been brought to light as the traditional testing methodologies do not adequately address the complexity of their risks on human health [4]. Therefore, complementing the uterotrophic assay with suitable molecular-level endpoint data could diminish the chances of dismissing potential estrogenic EDCs from further investigations because they do not meet statistical significance criteria for organ weight gain only, especially in studies involving a small number of animal subjects in the treatment groups. Such multiple-measure assessment that includes biological information could also justify further targeted studies to identify specific links between chemical interactions and toxicological effects. These targeted approaches could not only assist screening of the already narrowed down list of candidate EDCs but also be used in helping prioritize them for additional testing to assess their estrogenicity.

Transcriptome-level changes that take place early after exposure to 17β-estradiol (E_2_, the main human estrogen) and estrogenic EDCs have been investigated [16,17,18,19]. However, the focus of these studies has been on, e.g., the understanding of developmental changes in the uterus and the effects on fertility [20,21], or exploration of systemic effects due to environmental contaminants [22,23]. A limited number of reports are available about the identification of estrogen-regulated proteins in the rodent uterus using proteomics [24,25], and only one of them has presented tangible results focusing on uterine proteins regulated by E_2_ exposure in a mouse model [24]. Critical data in this regard have not been available for potential use in the context of the rat uterotrophic assay. Proteomics, the large-scale study of a proteome (proteins in a cell or tissue) of an organism, has become an increasingly mature methodology relying mostly on mass spectrometry and its advances [26,27,28,29,30,31,32]. Mass spectrometry-based proteomics has two branches: discovery-driven (or untargeted “shotgun”) proteomics and targeted proteomics [33], which is commonly performed sequentially. 

Here, we report the identification of proteins regulated by E_2_ using a quantitative proteomic investigation in the rat uterus first by an untargeted label-free discovery approach based on high resolution (Orbitrap) mass spectrometry (MS) and tandem mass spectrometry (MS/MS) in combination with online nanoflow liquid chromatography (LC). Then, verification of selected markers of estrogenicity is presented along with an exploratory application of targeted proteomics methods to bisphenol A (BPA) as a well-known example of estrogenic EDCs [34]. BPA represents 2,2-bis(4-hydroxyphenyl)propane, a synthetic chemical used to make polycarbonates and epoxy resins on an industrial scale. Biomonitoring studies show that human and animal exposure to BPA is rapid and continuous from the environment [34]. Altogether, this compound is a proven endocrine disruptor that mimics estrogens as a ligand at the cognate receptors thereby altering hormone concentrations and its metabolism. 

## 2. Results and Discussion

Discovery-driven proteomic analysis was performed using an adult OVX female rat model, as described for the testing of EDCs using the EPA’s uterotrophic assay [14,15]. We adapted a rapid tissue proteomics-based approach used previously for a quantitative survey of the impact of E2 on the OVX rat retina [35]. Using the Mascot database search algorithm within Proteome Discoverer (version 2.3), 2683 total protein identifications including 1547 high confidence protein identifications were obtained at the desired false discovery rate (FDR) set to ≤1%. However, only 774 proteins (representing 615 protein clusters) passed Scaffold’s rigorous validation criteria relying on both Peptide Prophet [36] and Protein Prophet [37] with at least two unique tryptic peptides identified for each protein (Table S1). For label-free quantification (LFQ), we used spectral counting (SC) [24,35] to detect uterine protein expression differences between the E2-treated versus vehicle control groups of OVX rats. Spectral counting is a quantitative method that counts and compares the number of fragment spectra identifying peptides of any protein. Although the technique has been capable of providing reasonable estimates about the extent of changes in protein abundances, they are generally not quantitatively accurate compared to targeted proteomics. Nevertheless, trends (i.e., up- and down-regulation of proteins) can be reliably identified through such label-free quantification [38]. Altogether, spectral counts of 165 proteins were affected significantly by E_2_ exposure of the uterus; among these 143 were up-regulated and 22 were down-regulated by the hormone (Table S2).

To advance from this list of potential markers of estrogenicity in the rat uterus towards targeted proteomics in the context of the uterotrophic assay, one may focus on selected findings considering previous reports. For example, several of the estrogen-regulated proteins, such as the vitamin D-dependent calcium-binding protein S100G and transglutaminase 2 (TGM2), have been previously reported from mouse transcriptomic studies [14,15,16]. However, our broader context justified comprehensive bioinformatics to guide the selection of targets. Therefore, we used Ingenuity Pathway Analysis (IPA^®^) to find functional interactions between the up- and down-regulated proteins identified in our study. Proteins found to be significantly affected by E_2_ in the OVX rat uterus mapped across ten heuristic IPA^®^ networks (Figures S1–S10). Associated functions of these networks included gene expression, ribonucleic acid (RNA) damage and repair, RNA post-transcriptional modification, protein synthesis, cellular assembly and organization, cellular compromise, drug metabolism, cell morphology, embryonic development, cellular function and maintenance, cellular growth, and proliferation, cancer, connective tissue disorder, etc.—all with relevance to estrogen biology shown, e.g., by the presence of an estrogen receptor (ER) or E_2_ (“beta-estradiol”) as a node in two specific networks (Figures S1 and S2). Although other networks were not associated with ER, non-genomic mechanisms of estrogen actions have been well documented and may operate through synergistic or additive effects involving the activation of ER signaling in the context of endocrine disruption [39]. Three networks also overlapped regarding the involved proteins and functions (Figure S11).

**Figure 1:**
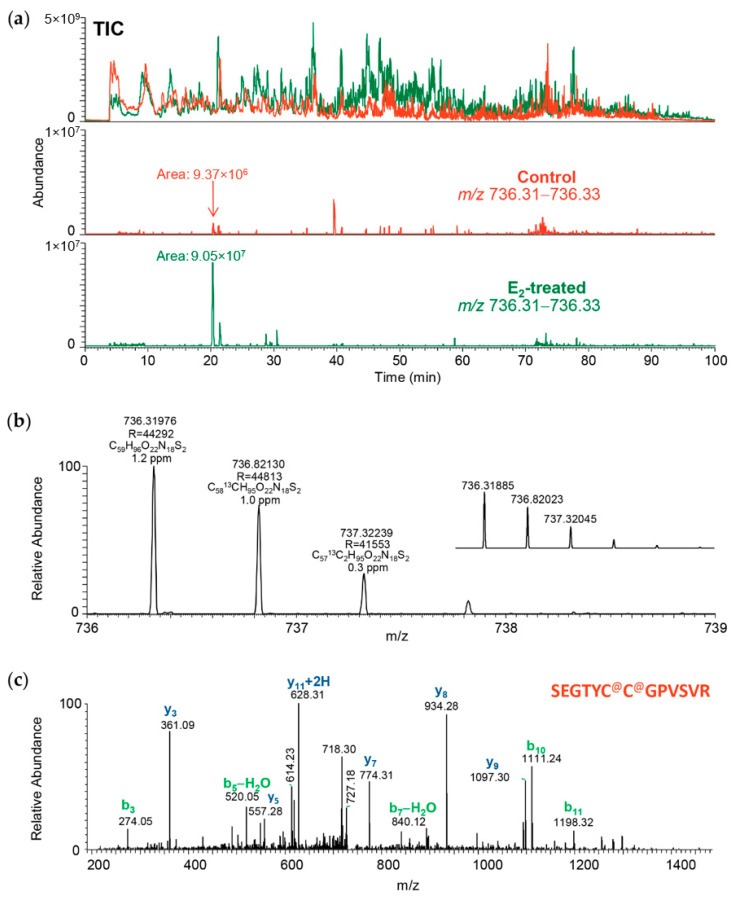
Screening from acquired high mass resolution (Orbitrap) mass spectra during the untargeted shotgun acquisitions for transglutaminase 2 (TGM2) as a prioritized uterine protein marker for targeted assay development. (**a**) Locating a proteotypic tryptic peptide of TGM2 by accurate mass of its doubly-charged ion [M+2H]^2+^ (TIC: total-ion chromatogram, brown and green traces: uterus samples from control and E2-treated rat, respectively); (**b**) Verifying accurate masses and isotope peak distribution of the doubly-charged ions (R: mass resolution, inset: theoretical m/z and isotope peak distribution); (**c**) Confirming the peptide sequence SEGTYC^@^C^@^GPVSVR by the acquired ion-trap tandem mass spectrometry (MS/MS) scan (b and y sequence ions marked according to nomenclature by Roepstorff and Fohlman [41]; C^@^ is carbamidomethylated cysteine).

**Figure 2:**
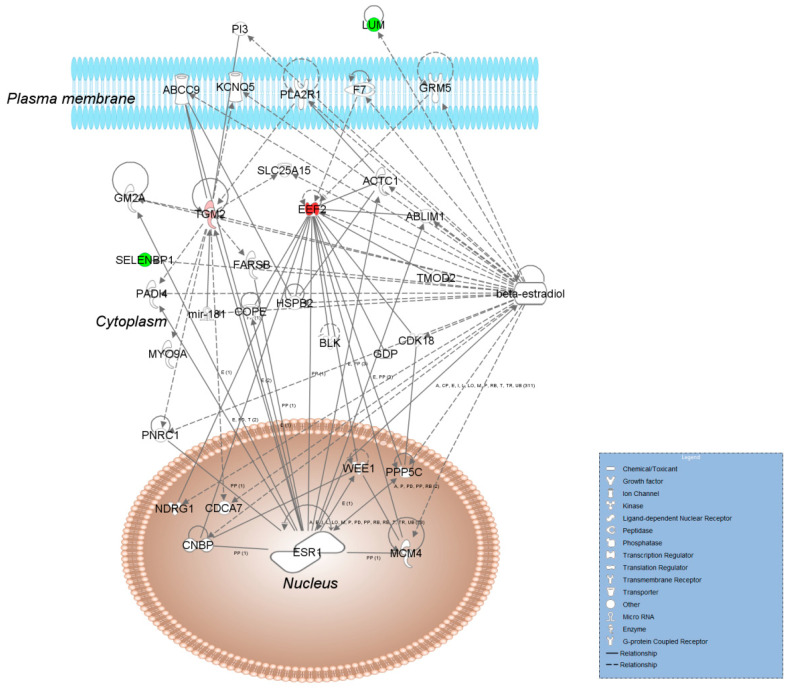
Molecular interaction Ingenuity Pathway Analysis (IPA^®^) network associated with connective tissue development and function, organ morphology, reproductive system development, and function assembled from the E_2_-regulated uterine proteins TGM2, elongation factor 2 (EEF2), selenium binding protein 1 (SELBP1), and lumican (LUM) in the rat. The shapes (see legend in the blue box) represent molecular classes of the regulated proteins. In the network, red and green colors denote upregulation and downregulation in response to E_2_ treatment, respectively. The intensity of the color indicates the relative magnitude of fold change in protein expression pattern based on spectral counts. Solid and dashed lines represent direct and indirect interactions, respectively. Abbreviations: ABLIM1, actin binding LIM protein 1; ABCC9, adenosine triphosphate (ATP) binding cassette subfamily C member 9; ACTC1, actin alpha cardiac muscle 1; BLK, proto-oncogene, Src family tyrosine kinase; CDCA7, cell division cycle associated 7; CDK18, cyclin dependent kinase 18; CNBP, CCHC-type zinc finger nucleic acid binding protein; COPE, COPI coat complex epsilon; ESR1, estrogen receptor 1; F7, coagulation factor 7; FARSB, phenylalanyl–tRNA synthetase subunit beta; GDP, guanosine diphosphate; GM2A, ganglioside activator; GRM5, glutamate metabotropic receptor 5; HSPB2, heat shock protein family B (small) member 2; KCNQ5, potassium voltage-gated channel subfamily Q member 5; MCM4, minichromosome maintenance complex component 4; mir, microRNA; MYO9A, myosin IXa; NDRG1, N-myc downstream regulated 1; PADI4, peptidyl arginine deiminase 4; PI3, peptidase inhibitor 3; PLAZR1, phospholipase A2 receptor 1; PNRC1, proline rich nuclear receptor coactivator; PPPP5C, protein phosphatase 5 catalytic subunit; SLC25A15, solute carrier family 25 member 15; TMOD2, tropomodulin 2; WEE1, G2 checkpoint kinase.

In addition, accurate (inclusion) mass screening [40] relying on the acquired raw data from the untargeted shotgun experiments was performed to support the selection of potential uterine markers from bioinformatic analyses involving the list of proteins found to be affected significantly by E_2_ exposure. This approach helped bridge high-resolution mass spectrometry-based untargeted discovery with the targeted assay development done typically by using multiple reaction monitoring (MRM) of peptides by LC-MS/MS on triple quadrupole instruments. The advantage of the high mass accuracy of the Orbitrap (>3 ppm for full-scan MS acquisition even without an internal “lock mass”) to verify peptides and sequences from an extensive list of candidate proteins is illustrated in Figure 1 and Figures S12–S14 for the selected four rat uterine proteins (upregulated and downregulated by E_2_ treatment, two proteins each, respectively). When these prioritized candidate proteins were assembled in a heuristic IPA^®^ network shown in Figure 2, associated functions included connective tissue development and function, organ morphology, reproductive system development, and function highly relevant to the uterotrophic activity of estrogen.

TGM2 is in a class of enzymes that catalyze the crosslinking of proteins by epsilon-gamma glutamyl lysine isopeptide bonds. This protein was shown to be activated in bovine uteruses and mouse livers [18,42]. TGM2, while being involved in Ca^2+^-dependent transamidation of proteins, is also involved in roles that are non-enzymatic and not Ca^2+^-dependent. TGM2 also participates in ATP and GTP hydrolysis, as well as in signal transduction through G-protein coupled receptors. Altogether, this protein potentially plays a critical role in remodeling the cytoskeletal structure and maintaining structural integrity, and variation in its abundance could contribute to the major morphological changes involved with the growth of the uterus upon E_2_ exposure. In addition to all these enzymatic roles, it also has multiple interactions with protein scaffolds [43]. In vivo using proteomics, TGM2 has not been identified previously as estrogen-regulated. Therefore, our finding is significant given the protein’s physiological importance and presence in the interaction networks we show in Figure 2 and Figure S8.

EEF2 belongs to the GTP-binding translational elongation factor family and serves as an important checkpoint regulator. It is essential for protein synthesis, as it catalyzes the GTP-dependent ribosomal translocation step in translation. EEF2 is a well-known up-regulated marker of estrogen exposure [24,44,45] shown previously in mouse uterus and cancer cells. Its expression in the E_2_-challenged uterus could be explained by the need for an increase in protein syntheses to keep up with the demands of the growth of the organ. This is consistent with our discovery data showing the majority of the estrogen-regulated proteins are activated.

SELENBP1, a protein that binds this trace elemental component of nonconventional amino acids selenocysteine and selenomethionine as well as selenoproteins, may be involved in the sensing of reactive xenobiotics in the cytoplasm and may be involved in intra-Golgi protein transport [46,47,48]. SELENBP1 expression has been shown by transcriptomics to be downregulated upon E_2_ exposure in the uterus and in cancer cells [17,49]. While its role is not fully understood, as a binding protein of selenium it contributes to the maintaining of the redox state of the cell by keeping free selenium concentrations low. Selenium is toxic to the cell in large concentrations yet it is necessary for the functioning of antioxidant selenoproteins. E_2_ exposure could cause a shift in the redox state of the cell and hence could cause a loss in the protective function of SELENBP1. This protein is another novel finding in our study as it has not been shown previously as estrogen-regulated in vivo in the rat uterus using proteomics.

LUM is in the family of small leucine-rich proteoglycans (SLRPs). This protein has been shown to be constitutively expressed and deposited in the extracellular matrix of the mouse uterus in the absence of the ovarian hormones but remodels along the estrous cycle and early pregnancy [50]. LUM modulates synthesis, deposition, and degradation of various molecules, and has been found among the proteins downregulated by E_2_ treatment in our earlier proteomics study of the mouse uterus [24]. Overall, its biological role and previous experimental evidence have justified the inclusion of this protein as a prioritized candidate for targeted assay development for estrogenic ECDs.

The developed targeted assay for TGM2, EEF2, SELENBP1, and LUM using heavy-isotope labeled peptides as internal standards are summarized in Table S3. Targeted proteomics was recognized as the method of the year in 2012 [51,52]. It is a specific and selective way to assay proteins of interest. Adapted from small-molecule methodologies and applied to targeted protein analyses, monitoring only selected proteotypic peptides facilitates an increase in sensitivity, reproducibility, as well as in specificity, and robustness when applied to a complex mixture of peptides obtained from a proteomics workflow. We developed the LC-MRM method for use with conventional high-performance liquid chromatography (HPLC, column i.d. of 2.1 mm) to facilitate adaptation by laboratories not equipped with nanoflow LC capabilities.

**Figure 3:**
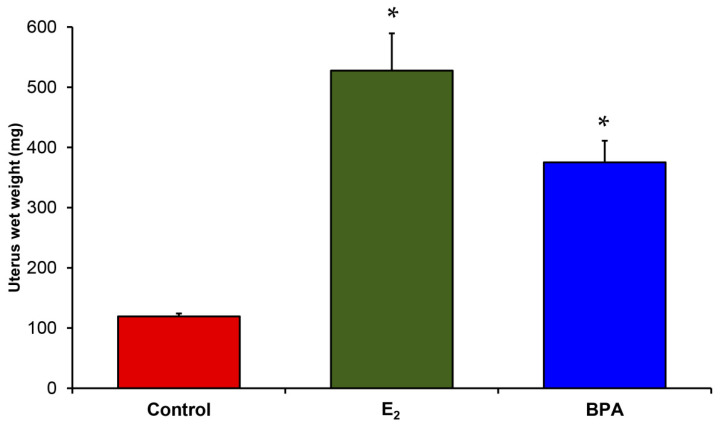
Uterus wet weights of E_2_-treated and BPA-treated OVX Sprague-Dawley rats compared to vehicle-treated controls in the reported experiment. Data are given as average ± standard error, and asterisks (*) indicate statistically significant differences from OVX controls with ANOVA followed by post hoc Dunnett’s tests (*p* < 0.05).

We used the developed targeted proteomics assays for relative quantification in a study applied to BPA as a well-known example of estrogenic ECDs [34] using E_2_ treatment as a positive control. BPA-based polymers such as polycarbonate plastics and epoxy resins are widely used [34]. BPA is a weak estrogenic EDC and has been a major target for endocrine disruption-related studies [53,54]. BPA can leach from plastic products and expose humans and the environment to its potentially harmful effects as an EDC [55,56,57,58]. Public concern about BPA exposure is so great because of its widespread use, and has led to a movement to specifically label products made without it as “BPA-free.” As shown in Table 1, our targeted proteomics method allowed for determining quantitative changes of the chosen E_2_-regulated proteins in the rat uterus based on their proteotypic tryptic peptides across samples. Overall, changes in the expression of TGM2, EEF2, SELENBP1, and LUM replicated the trend observed from the wet uterus weight-based assessment displayed in Figure 3.

In conclusion, we presented differential proteome analyses focusing on estrogen-regulated rat uterus proteins using quantitative proteomics approaches: first a label-free shotgun method followed by targeted quantitation as a roadmap for the complementation of the conventional uterotrophic assay. To our knowledge, this is the first study to identify and verify in vivo rat uterine protein markers for potential screening of candidate EDCs regarding effects that mimic those of estrogen.

## 3. Materials and Methods

### 3.1. Chemicals

All HPLC grade solvents were obtained from Fisher Scientific (Atlanta, GA, USA). Sequencing grade trypsin was from Applied Biosystems (Foster City, CA, USA). All other chemicals were acquired through Sigma-Aldrich (St. Louis, MO, USA) unless otherwise stated.

### 3.2. Animals

Sprague Dawley rats weighing 200−250 g were obtained from Charles Rivers Laboratories (Wilmington, DE, USA). They were kept under a standard 12 h light/12 h dark cycle, and the room temperature was maintained at 21 °C. Ovariectomy of the rats was done by the supplier (Charles River Laboratories). Animals were shipped approximately one week after the procedure and allowed to adapt in the animal facility for approximately two weeks before starting their treatment. Two animals were housed per cage with full access to standard diet and water.

For discovery-driven proteomics, daily subcutaneous (s.c.) injections with the vehicle control (corn oil, 60 µL per injection), or E2 (50 µg/kg body weight in corn oil vehicle) were done on 5 consecutive days between 10:00 a.m. and 12:00 a.m., and the rats were killed for immediate tissue collection on the sixth day after starting the experiments. The targeted proteomics experiments were done using a separate cohort of animals with treatments identical as above and with an added group of OVX rats that received s.c. injections of BPA (300 mg/kg body weight in corn oil vehicle). Except for the BPA group (N = 5), each treatment group consisted of four animals. The animals were sacrificed by cervical dislocation, decapitated, and their brains were removed. An abdominal incision was then made and the uterus was removed by cutting at the junction of the uterus and vagina and at the site of the ovariectomy on each horn. Excess fat and connective tissues were removed, and the organ was blotted and weighed. All tissues were stored at –80 °C until sample preparation and analysis.

### 3.3. Sample Preparation

Tissues were incubated in 200 µL of 8 M urea for 30 min, as reported before [24,35]. The samples were centrifuged for 5 min at 1400× *g* and the supernatant was collected. The protein content of uterine extracts was determined by a microBCA assay (Bio-RAD, Hercules, CA, USA). Approximately 100 µg of protein from each sample was used for further processing. Samples were reduced with 1 mM dithiothreitol (DTT) for 30 min at 65 °C to reduce the disulfide bonds. Carbamidomethylation of the thiol groups was performed by the addition of 5 mM iodoacetamide (IAA) and incubation for 30 min at room temperature in the dark. Excess IAA was quenched by the addition of DTT for 5 min. The samples were diluted with 50 mM ammonium bicarbonate to lower the urea concentration to less than 2 M. Samples were digested with sequencing grade trypsin (1:50, Applied Biosystems, Foster City, CA) overnight. The tryptic digestion was terminated by acidifying the samples to pH < 2.0 with acetic acid and the digests were desalted using C18 Sep-Pak solid-phase extraction cartridges (Waters, Milford, MA, USA). The desalted uterine tryptic digests were further dried under vacuum (Vacufuge^TM^, Eppendorf AG, Hamburg, Germany) and subsequently reconstituted in 20 µL of 5% (*v*/*v*) acetonitrile in water containing 0.1% (*v/v*) acetic acid and aliquots of 5 µL were used for LC-MS/MS analyses.

### 3.4. Data-Dependent LC-MS/MS Data Acquisition for Discovery-Driven Shotgun Proteomics

The digested samples were analyzed using a hybrid ion trap–Orbitrap tandem mass spectrometer (LTQ Velos Orbitrap Pro) coupled to an EASY nLC-1000 nanoflow liquid chromatography system fitted with a 15 cm × 75 μm i.d. EasySpray column packed with 3 µm PepMap C18 particles (Thermo Fisher Scientific, San Jose, CA, USA) [35]. Gradient elution was used: solvent A and solvent B were water and acetonitrile, respectively, and each contained 0.1% (*v*/*v*) formic acid. Samples (equivalent to 100 µg protein) were reconstituted in 100 µL of the solvent containing 5% (*v*/*v*) acetonitrile and 0.1% (*v*/*v*) formic acid in water and transferred into 200 µL polypropylene autosampler vials closed with an open-top screw cap and Teflon-lined silicon septum (USA Scientific, Orlando, FL, USA). During a 20-min column equilibration at 5% B, 5 µL of the solution was injected while maintaining constant column pressure at 600 bar. The peptides were eluted at 300 nL/min using the following gradient: (1) 5 min isocratic at 5% B; (2) linear program to 40% B over 90 min and then (3) isocratic at 40% B for 5 min; (4) to 90% B over 5 min; (6) isocratic at 90% B for 5 min; and (6) resetting to 5% B in 20 min. The mass spectrometer was operated in positive-ion nanoelectrospray (nanoESI) mode with a source voltage of 2.0 kV and ion-transfer tube temperature of 275 °C. Full-scan mass spectra were acquired at 60,000 resolution (*m/z* 400) in the Orbitrap and up to 20 MS/MS spectra were obtained in the ion trap for each full spectrum acquired using collision-induced dissociation (CID) of multiply-charged ions (z ≥ 2). Dynamic exclusion was set for 60 s after an ion was selected for fragmentation. Two technical replicates were run for each sample.

### 3.5. Database Search, Label-Free Relative Quantification, and Pathway Analysis

MS/MS spectra were searched against the UniProt protein sequence database (species: *Rattus norvegicus*, 29,938 entries) using the Mascot search engine (version 2.6.2; Matrix Science, Boston, MA, USA) run from Proteome Discoverer (version 2.3; Thermo Fisher Scientific). A parent ion mass tolerance and fragment ion mass tolerance were set to 25 ppm and 0.80 Da, respectively, allowing only one missed cleavage in our search filters and limiting FDR to 0.01 (1%). Cystein carbamidomethylation was indicated as fixed modification and methionine oxidation was designated as variable modification. We used Scaffold software (version 4.9.0, Proteome Software Inc.; Portland, OR, USA) to validate our search results using the Peptide Prophet [36] and Protein Prophet [37] algorithms requiring over 95% and 99% probabilities, respectively, and at least two identified unique peptides for each protein. Our LFQ relied on SC [23] built into the Scaffold software, and *p* < 0.05 was considered significantly different using unpaired *t*-tests for statistical comparison between sample categories. We also considered a twofold change in spectral counts as a threshold of biological effect. Missing values, if any, were handled using Scaffold’s default method and settings. The identified E_2_-regulated proteins were submitted to Ingenuity Pathway Analysis^®^ (IPA^®^, QIAGEN, Redwood City, CA, USA; https://www.qiagenbioinformatics.com/products/ingenuity-pathway-analysis/) (accessed on 12 January 2021) to derive bioinformatics annotations along with potential protein interaction networks, as well as associated biological functions and processes. Overlaps of *p*-values were reported from IPA^®^’s calculations using the right-tailed Fisher’s exact test.

### 3.6. Target Panel and Internal Standards

Two proteotypic peptides were selected and subjected to BLASTP analysis for each protein (TGM2, EEF2, SELENBP1, and LUM) to confirm 100% homology. The selected peptides did not have any missed cleavages and did not have extensive post-translational modifications. Carbamidomethylated cysteine was incorporated as a fixed modification in all peptides when required. The approach involved the use of stable-isotope labeled internal standards (SIS) added to the tissue extracts for comparison against native tryptic peptide (NAT) levels in the digested samples. The SIS peptides were purchased from New England Peptide (Gardner, MA, USA). Incorporation of the ^13^C- and ^15^N-isotopes was done at the C-terminal residue of tryptic peptides yielding mass shifts of +8 Da (from ^13^C_6_^15^N_2_-lysine), +10 Da (from ^13^C_6_^15^N_4_-arginine), and +7 Da (from ^13^C_6_^15^N-leucine) compared to their unlabeled counterparts in the samples.

### 3.7. MRM-Based LC–MS/MS Data Acquisition for Targeted Proteomics

Samples were analyzed in triplicate using a TSQ Quantum Ultra tripe-quadrupole mass spectrometer (TSQ, Thermo Electron Corporation, Trace Chemical Analysis, Austin, TX, USA) equipped with a heated electrospray ionization (H-ESI) source and operated with Xcalibur (version 2.2) and Tune Plus (version 2.2) data acquisition software.

Gradient HPLC separations were carried out using a Surveyor MS solvent delivery system (Thermo). The Phenomenex (Torrance, CA, USA) Aeris™ PEPTIDE XB-C18 column (15 cm × 2.1 mm i.d., packed with 3.6-μm core-shell particles) was operated at 0.4 mL/min flow rate and with the following gradient program: 2% B to 65% B in 30 min, then ramped to 95% B in 0.5 min and held for 3.5 min, and finally, the column was ramped to 2% B in 0.5 min and equilibrated for 10.5 min. The autosampler injection volume was 10 μL, and the tray temperature was maintained at 18 °C. H-ESI spray voltage, H-ESI temperature, and capillary temperature were 3.5 kV, 275 °C, and 300 °C, respectively. Nitrogen sheath gas and auxiliary gas flow rates were 30 and 20 arbitrary units (corresponding to approximately 0.45 and 6.0 L/min according to the manufacturer’s specification), respectively. CID was performed with argon at 1.5 mTorr pressure. MRM with a unit mass resolution for the precursor and product ions was used for the quantitation of peptides. Data acquisition and processing were controlled by the XCalibur software (version 2.1) of the instrument. A complete list of the MRM transitions used in this study is provided in Supplementary Table S3.

### 3.8. Data Processing for Targeted Proteomics and Statistical Analysis of the Results

Manual verification of the extracted ion chromatogram peak selections was performed with XCalibur software (version 2.1). The area under the curve (AUC) of the relative abundance of each peak for each transition was calculated from within the Xcalibur software. Relative quantitation was done by first taking the average from the technical replicates of all samples for each peptide, and then using the ratio of NAT to SIS peptide, comparing controls and treated samples to get fold change increase or decrease. Relative ratios of SIS internal standards to endogenous peptide, for each peptide, were normalized to the control to get fold change differences.

Statistical analysis of the relative fold change of peptides was performed using ANOVA followed by post hoc Dunnett’s tests. *p* < 0.05 was considered statistically significant.

## Figures and Tables

**Table 1:** E_2_-regulated proteins in OVX rat uterus verified by targeted proteomics and applied to testing BPA [34,53,54,55,56,57,58] as a well-known estrogenic EDC.

Rat Uterus Protein	Proteotypic Tryptic Peptide	Fold Change after E_2_ Treatment ^1^	Fold Change after BPA Treatment ^1^
Transglutaminase 2, C polypeptide (TGM2)	YSGCLTESNLIK	24.7 ± 4.6	14.3 ± 4.4
	SEGTYCCGPVSVR	17.5 ± 2.3	10.2 ± 2.1
Elongation factor 2 (EEF2)	EGIPALDNFLDKL	3.9 ± 0.3	3.2 ± 0.3
	TFCQLILDPIFK	8.4 ± 1.1	6.5 ± 1.1
Selenium-binding protein 1 (SELENBP1)	HEIIQTLQMK	–2.2 ± 0.5	–2.0 ± 0.2
	LILPSIISSR	–2.9 ± 0.1	–2.3 ± 0.2
Lumican (LUM)	NNQIDHIDEK	–3.8 ± 0.8	–6.3 ± 0.8
	SLEYLDLSFNQMSK	–2.0 ± 0.4	–2.5 ± 0.1

^1^ All reported results (data given as average ± standard error) yielded statistically significant differences from OVX controls with analysis of variance (ANOVA) followed by post hoc Dunnett’s tests, *p* < 0.05.

## Data Availability

The mass spectrometry proteomics data have been deposited to the ProteomeXchange Consortium via the PRIDE [59] partner repository with the dataset identifier PXD023273 and doi:10.6019/PXD023273.

## Supplementary Materials

The Supplementary Materials are available online at https://www.mdpi.com/1422-0067/22/4/1686/s1.
