# Supplementary material for: Proteomics Complementation of the Rat Uterotrophic Assay for Estrogenic Endocrine Disruptors: A Roadmap of Advancing High Resolution Mass Spectrometry-Based Shotgun Survey to Targeted Biomarker Quantifications
Source: Int J Mol Sci. 2021 Feb 8;22(4):1686. doi: 10.3390/ijms22041686 (PMC7914934; doi:10.3390/ijms22041686)
Supplement: Supplementary file 1 [file ijms-22-01686-s001.zip › ijms-1078409_FiguresS1-S14.pdf]

(**a**)

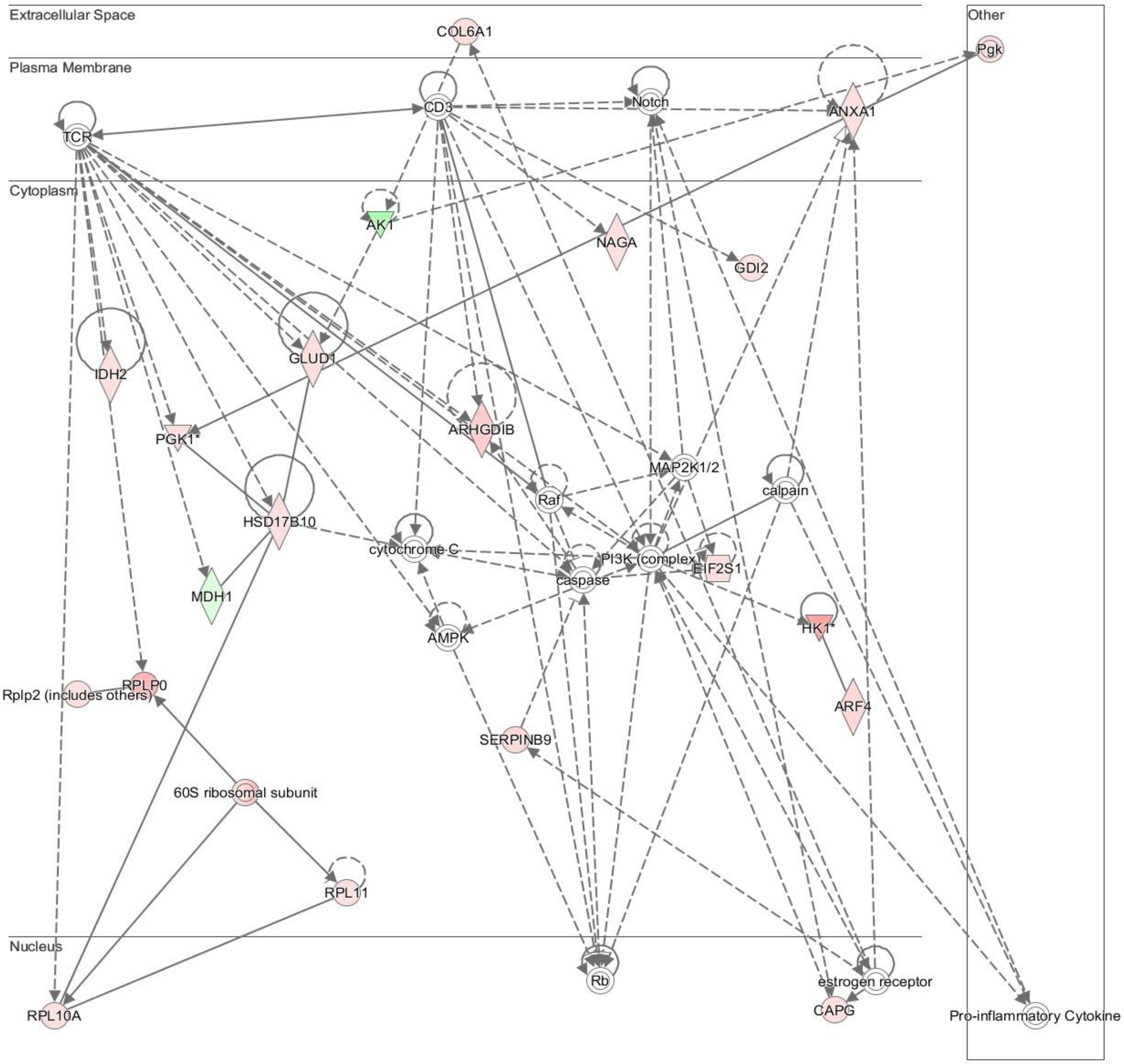

(**b**)

Canonical pathway	Target molecules	Z-score	P-value
Estrogen receptor signaling	AMPK, CYTOCHROME c, estrogen receptor, MAP2K1/2, notch, PI3 (complex), Pro-inflammatory cytokine, Raf	0.45	2.35E-03
Sirtuin signaling pathway	AMPK, cytochrome C, GLUD1, IDH2, PGK. PGK1, Pro-inflammatory cytokine	N/A	4.7E-03

Figure S1. IPA® network linked to cancer, gastrointestinal disease and protein synthesis (**a**). The network was assembled from E_2-regulated proteins in the uterus of OVX rats through identification by label-free shotgun proteomics. Red symbols: upregulated, green symbols: downregulated by the hormone; solid line: direct relationship, dashed line: indirect relationship. For further details on symbols and relationships, see http://qiagen.force.com/KnowledgeBase/articles/Basic_Technical_Q_A/Legend. (**b**) Top canonical pathways and their corresponding molecular targets associated with the network shown. Z-scores indicate activation or suppression of the corresponding pathway (positive or negative values, respectively; N/A: no prediction can be made).

(a)

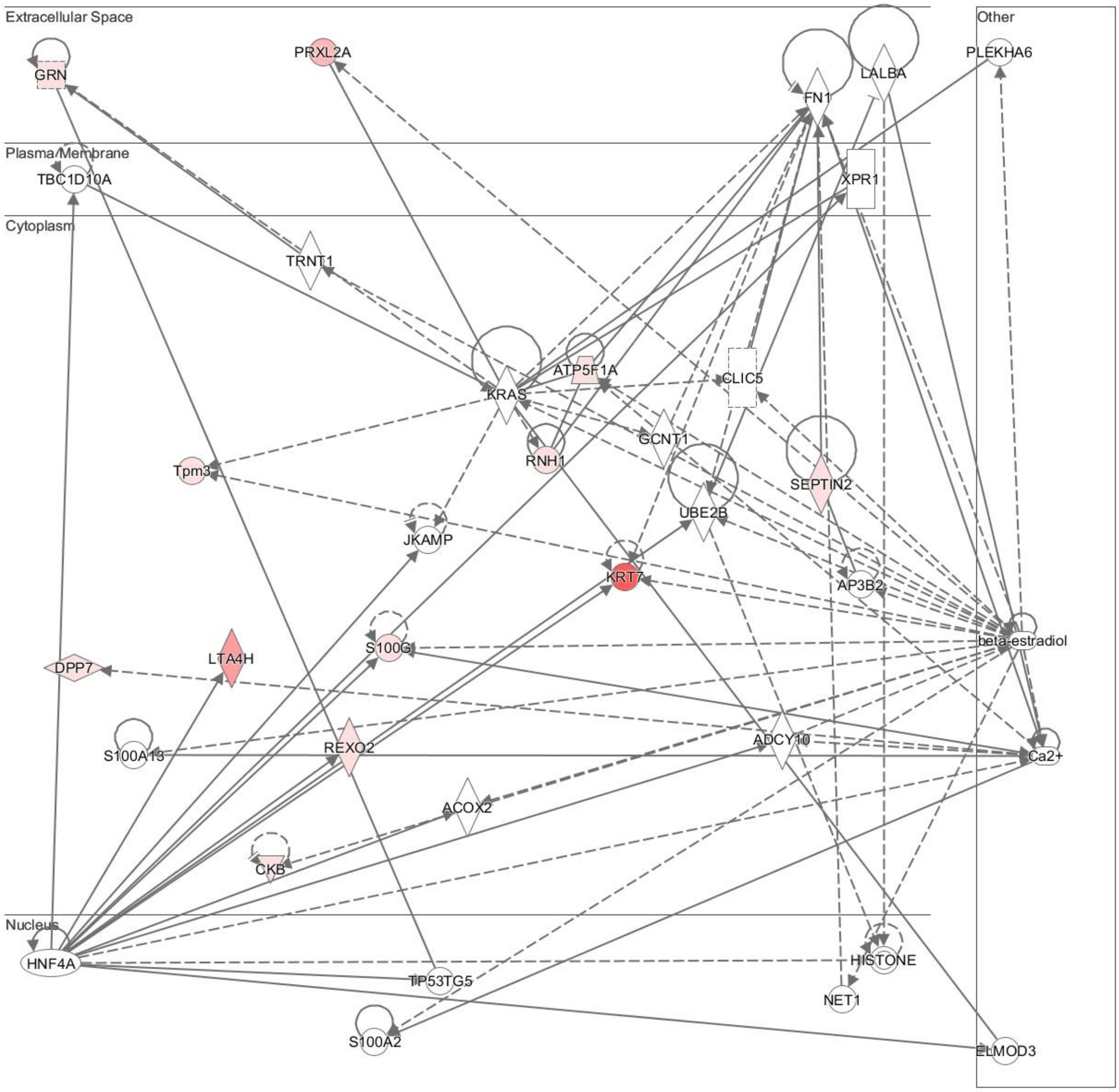

(b)

Canonical pathway	Target molecules	Z-score	P-value
Estrogen receptor signaling	ADCY10, ATP5F1A, Ca^{2+}, KRAS	0.45	2.35E-03
Glucocorticoid receptor signaling	ATP5F1A, Ca^{2+}, KRAS, KRT7	N/A	1.04E-06

Figure S2. IPA® network linked to cancer, cell to cell signaling and interaction, organismal injury and abnormalities (**a**). The network was assembled from E_2-regulated proteins in the uterus of OVX rats through identification by label-free shotgun proteomics. Red symbols: upregulated, green symbols: downregulated by the hormone; solid line: direct relationship, dashed line: indirect relationship. For further details on symbols and relationships, see http://qiagen.force.com/KnowledgeBase/articles/Basic_Technical_Q_A/Legend. (**b**) Top canonical pathways and their corresponding molecular targets associated with the network shown. Z-scores indicate activation or suppression of the corresponding pathway (positive or negative values, respectively; N/A: no prediction can be made).

(a)

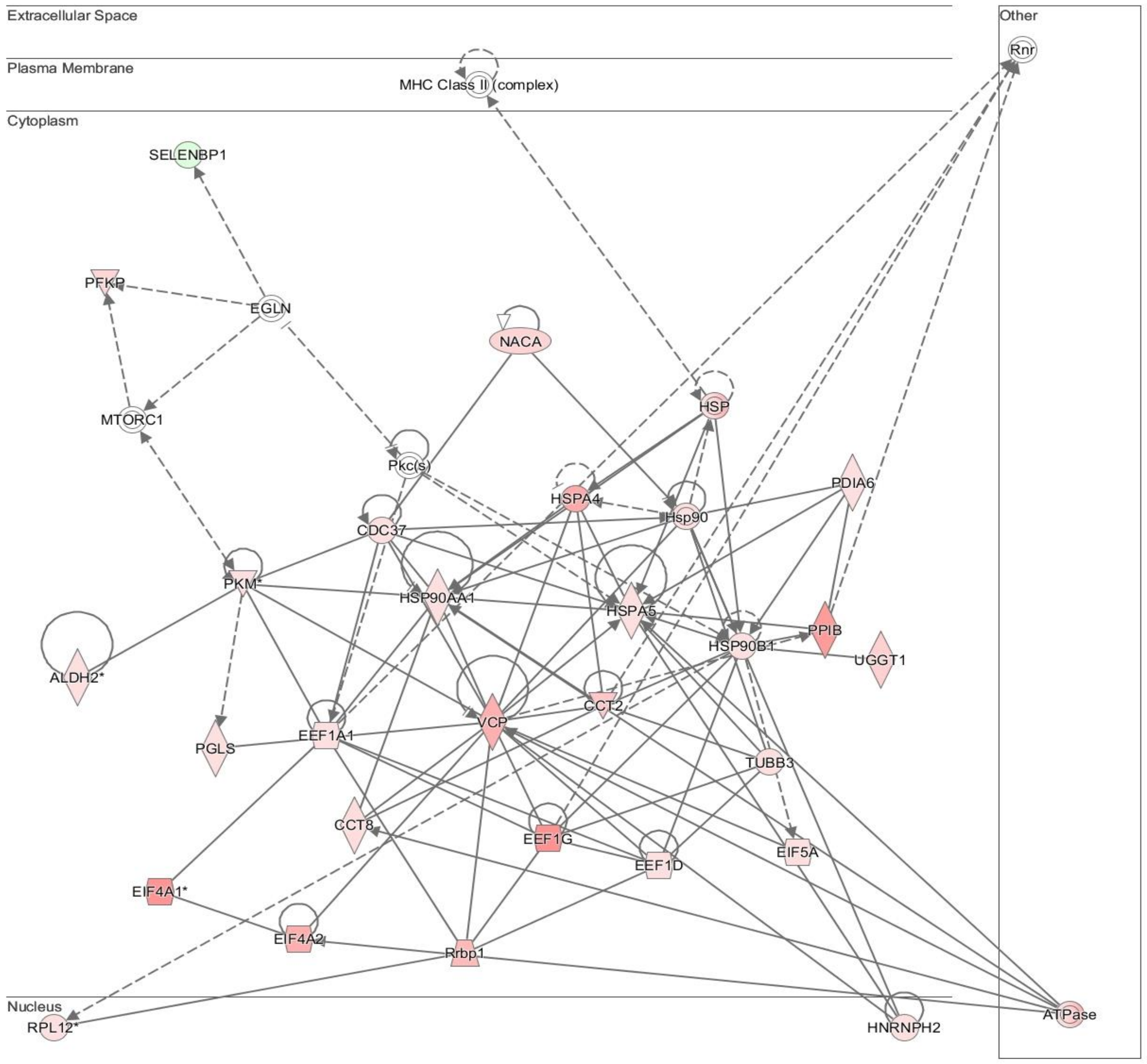

(**b**)

Canonical pathway	Target molecules	Z-score	P-value
Protein ubiquitination pathway	ATPase, HSP90, HSP,HSP90AA1,HSP90B1,HSPA4,HSPA5, MHC ClassII	N/A	5.79E-07
Estrogen receptor signaling	ATPase, HSP90, HSP,HSP90AA1,HSP90B1,MTORC1, Pkc(s)	0.45	2.35E-03

Figure S3. IPA® network linked to post-translational modification, protein folding and protein synthesis (**a**). The network was assembled from E_2-regulated proteins in the uterus of OVX Brown Norway rats through identification by label-free shotgun proteomics. Red symbols: upregulated, green symbols: downregulated by the hormone; solid line: direct relationship, dashed line: indirect relationship. See http://qiagen.force.com/KnowledgeBase/articles/Basic_Technical_Q_A/Legend for further details on symbols and relationships. (**b**) Top canonical pathways and their corresponding molecular targets associated with the network shown. Z-scores indicate activation or suppression of the corresponding pathway (positive or negative values, respectively; N/A: no prediction can be made).

(a)

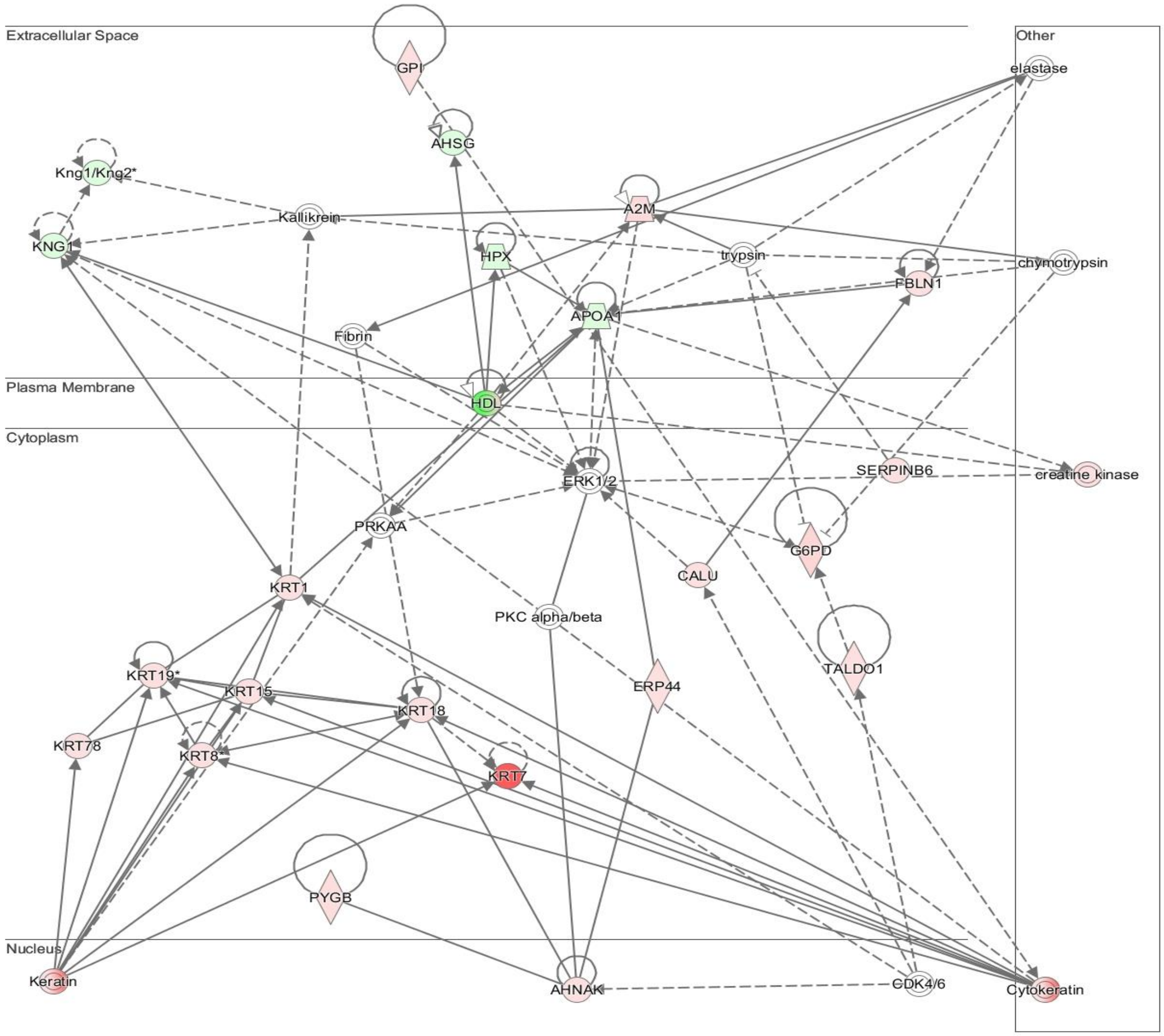

(**b**)

Canonical pathway	Target molecules	Z-score	P-value
PPARa/RXRa activation	APOA1, creatinine kinase, erk1/2, HDL, PKC alpha/beta, PRKAA	-2	4.67E-02
Estrogen receptor signaling	Elastase, ERK1/2, HDL, PKC alpha/beta, PRKAA	0.45	2.35E-03

Figure S4. IPA® network linked to cell morphology and embryonic development (**a**). The network was assembled from E_2-regulated proteins in the uterus of OVX rats through identification by label-free shotgun proteomics. Red symbols: upregulated, green symbols: downregulated by the hormone; solid line: direct relationship, dashed line: indirect relationship. For further details on symbols and relationships, see http://qiagen.force.com/KnowledgeBase/articles/Basic_Technical_Q_A/Legend. (**b**) Top canonical pathways and their corresponding molecular targets associated with the network shown. Z-scores indicate activation or suppression of the corresponding pathway (positive or negative values, respectively; N/A: no prediction can be made).

(a)

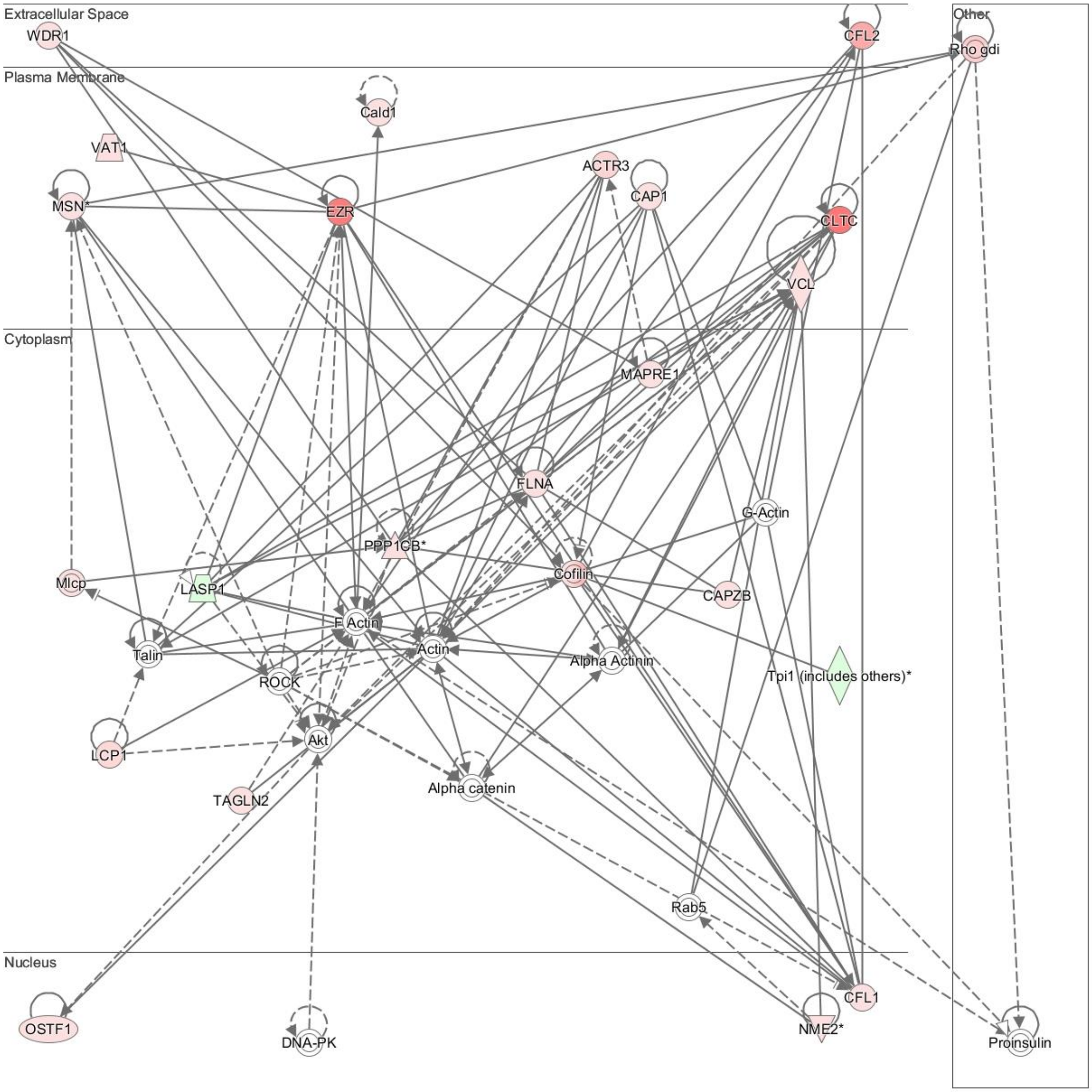

(**b**)

Canonical pathway	Target molecules	Z-score	P-value
Epithelial adherens junction signaling	Actin, actr3, Akt, alpha actinin, alpha catenin, F actin, G actin, VCL	N/A	1.31E-07
Estrogen receptor signaling	Akt, CFL1, CFL2, Cofilin, DNA-PK, Mlcp, PPP1CB, ROCK	0.45	2.35E-03

Figure S5. IPA® network linked to cell morphology, cellular assembly and organization, function and maintenance (**a**). The network was assembled from E_2-regulated proteins in the uterus of OVX rats through identification by label-free shotgun proteomics . Red symbols: upregulated, green symbols: downregulated by the hormone; solid line: direct relationship, dashed line: indirect relationship. See http://qiagen.force.com/KnowledgeBase/articles/Basic_Technical_Q_A/Legend for further details on symbols and relationships. (**b**) Top canonical pathways and their corresponding molecular targets associated with the network shown. Z-scores indicate activation or suppression of the corresponding pathway (positive or negative values, respectively; N/A: no prediction can be made).

(a)

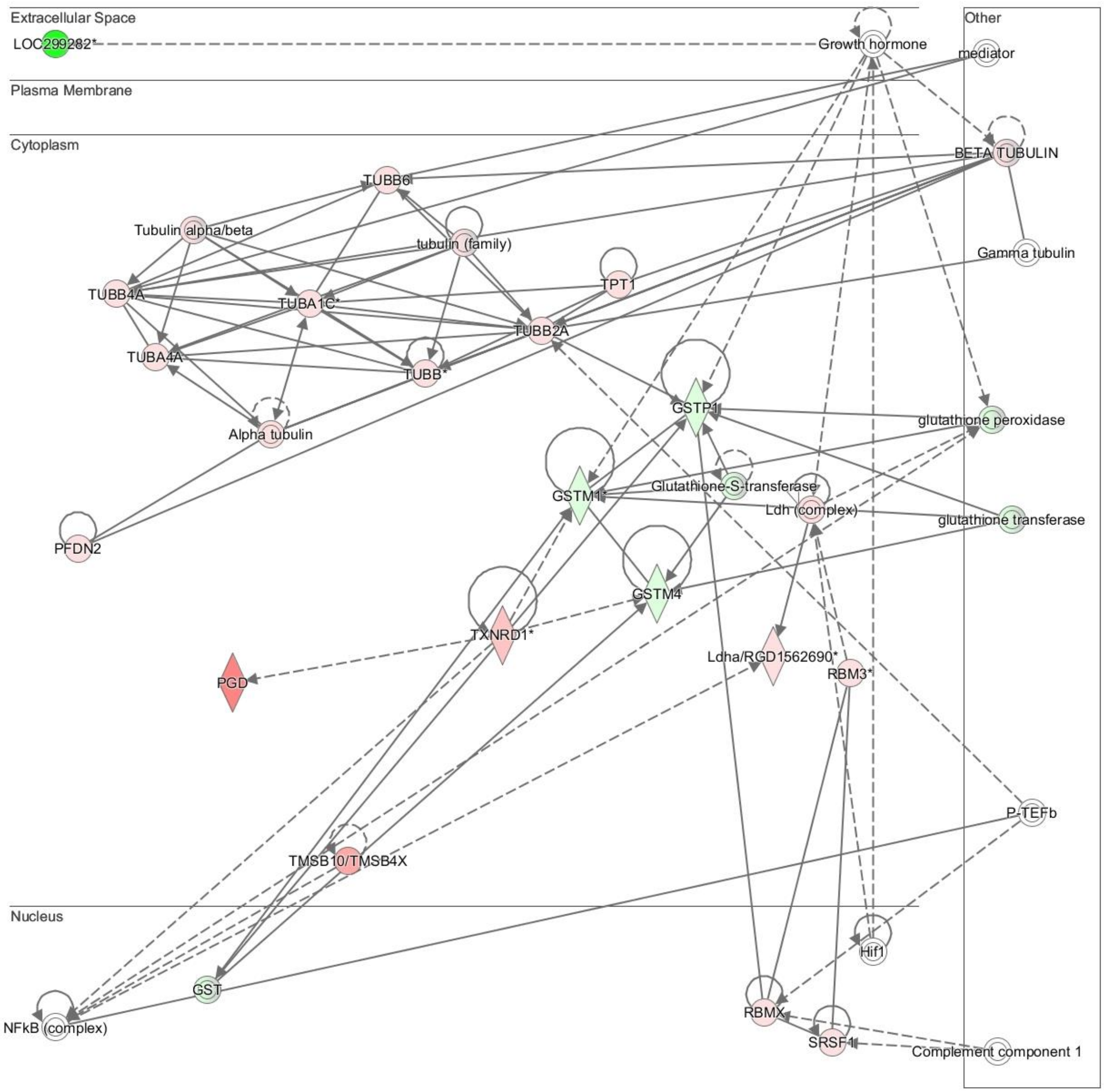

(**b**)

**Canonical pathway**	**Target molecules**	**Z-score**	**P-value**
Epithelial adherens junction signaling	Alpha tubulin, beta tubulin, gamma tubulin, TUBA1C, TUBA4A, TUBB6, TUBB, TUBB2A,TUBB4A, Tubulin (family), tubulin alpha/beta	N/A	1.31E-07
14-3-3 mediated signaling	Alpha tubulin, beta tubulin, gamma tubulin, TUBA1C, TUBA4A, TUBB6, TUBB, TUBB2A, TUBB4A, Tubulin (family), tubulin alpha/beta	N/A	3.36E-05

Figure S6. IPA® network linked to cancer, endocrine system disorder, organismal injury and abnormalities (**a**). The network was assembled from E_2-regulated proteins in the uterus of OVX rats through identification by label-free shotgun proteomics. Red symbols: upregulated, green symbols: downregulated by the hormone; solid line: direct relationship, dashed line: indirect relationship. For further details on symbols and relationships, see http://qiagen.force.com/KnowledgeBase/articles/Basic_Technical_Q_A/Legend. (**b**) Top canonical pathways and their corresponding molecular targets associated with the network shown. Z-scores indicate activation or suppression of the corresponding pathway (positive or negative values, respectively; N/A: no prediction can be made).

(a)

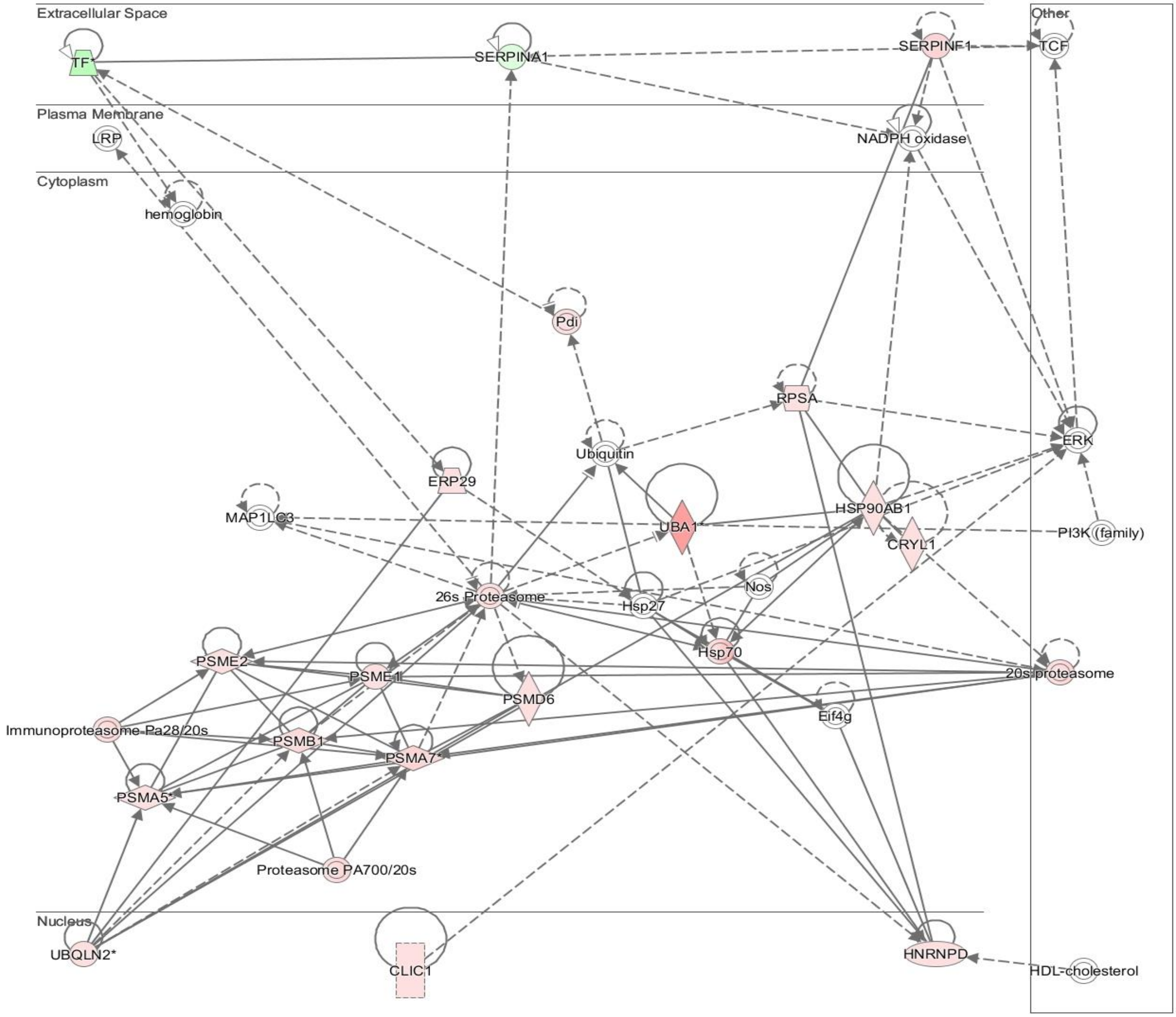

(**b**)

Canonical pathway	Target molecules	Z-score	P-value
Protein ubiquitination pathway	20s proteasome, 26s proteasome, Hsp27, HSP70, HSP90AB1, Immunoproteasome,Pa28/20S, Proteasome PA700/20s, PSMA5*, PSAM7*,PSMB1, PSMD6, PSME1,PSME2, uba1, ubiquitin	Na	5.79E-07
Estrogen receptor signaling	ERK, HDL-cholesterol. HNRNPD, HSP90AB1, Nos, P13K (family)	0.45	2.35E-03

Figure S7. IPA® network linked to gene expression, RNA damage and repair, and RNA post-transcriptional modification (**a**). The network was assembled from E_2-regulated proteins in the uterus of OVX rats through identification by label-free shotgun proteomics. Red symbols: upregulated, green symbols: downregulated by the hormone; solid line: direct relationship, dashed line: indirect relationship. For further details on symbols and relationships, see http://qiagen.force.com/KnowledgeBase/articles/Basic_Technical_Q_A/Legend. Inset table (**b**): Top canonical pathways and their corresponding molecular targets associated with the network shown. Z-scores indicate activation or suppression of the corresponding pathway (positive or negative values, respectively; N/A: no prediction can be made).

(a)

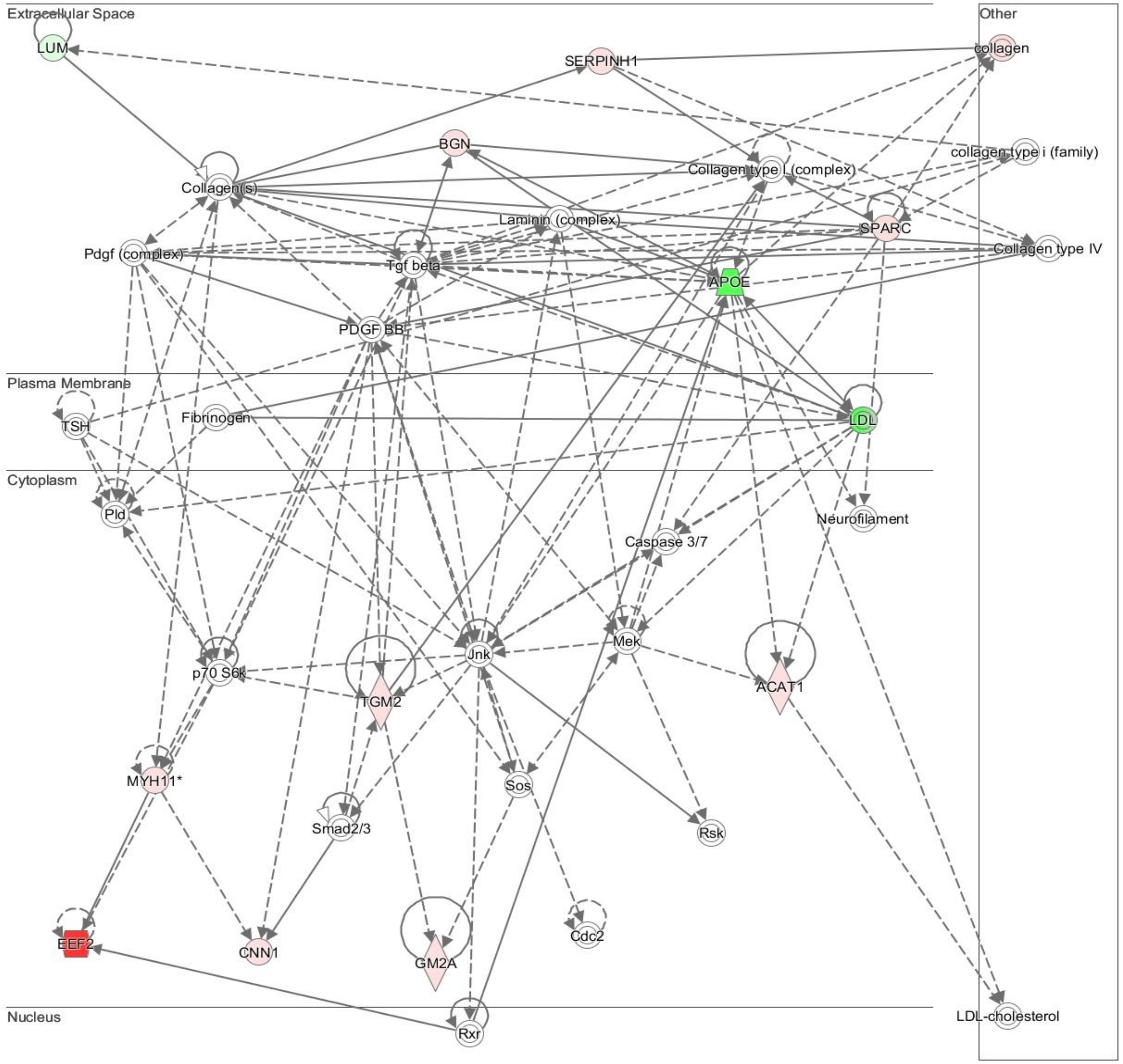

(**b**)

Canonical pathway	Target molecules	Z-score	P-value
PPARa/RXRa activation	Jnk, LDL, Mek, Rxr, Smad2/3, Sos, Tgf beta	-2	4.67E-02
Glucocorticoid receptor signaling	Fibrinogen, Jnk, Mek, Rsk, Rxr, Smad2/3, Sos, Tgf beta	N/A	1.04E-06

Figure S8. IPA® network linked to metabolic disease, organismal injury and abnormalities (**a**). The network was assembled from E_2-regulated proteins in the uterus of OVX rats through identification by label-free shotgun proteomics. Red symbols: upregulated, green symbols: downregulated by the hormone; solid line: direct relationship, dashed line: indirect relationship. For further details on symbols and relationships, see http://qiagen.force.com/KnowledgeBase/articles/Basic_Technical_Q_A/Legend. (**b**) Top canonical pathways and their corresponding molecular targets associated with the network shown. Z-scores indicate activation or suppression of the corresponding pathway (positive or negative values, respectively; N/A: no prediction can be made).

(a)

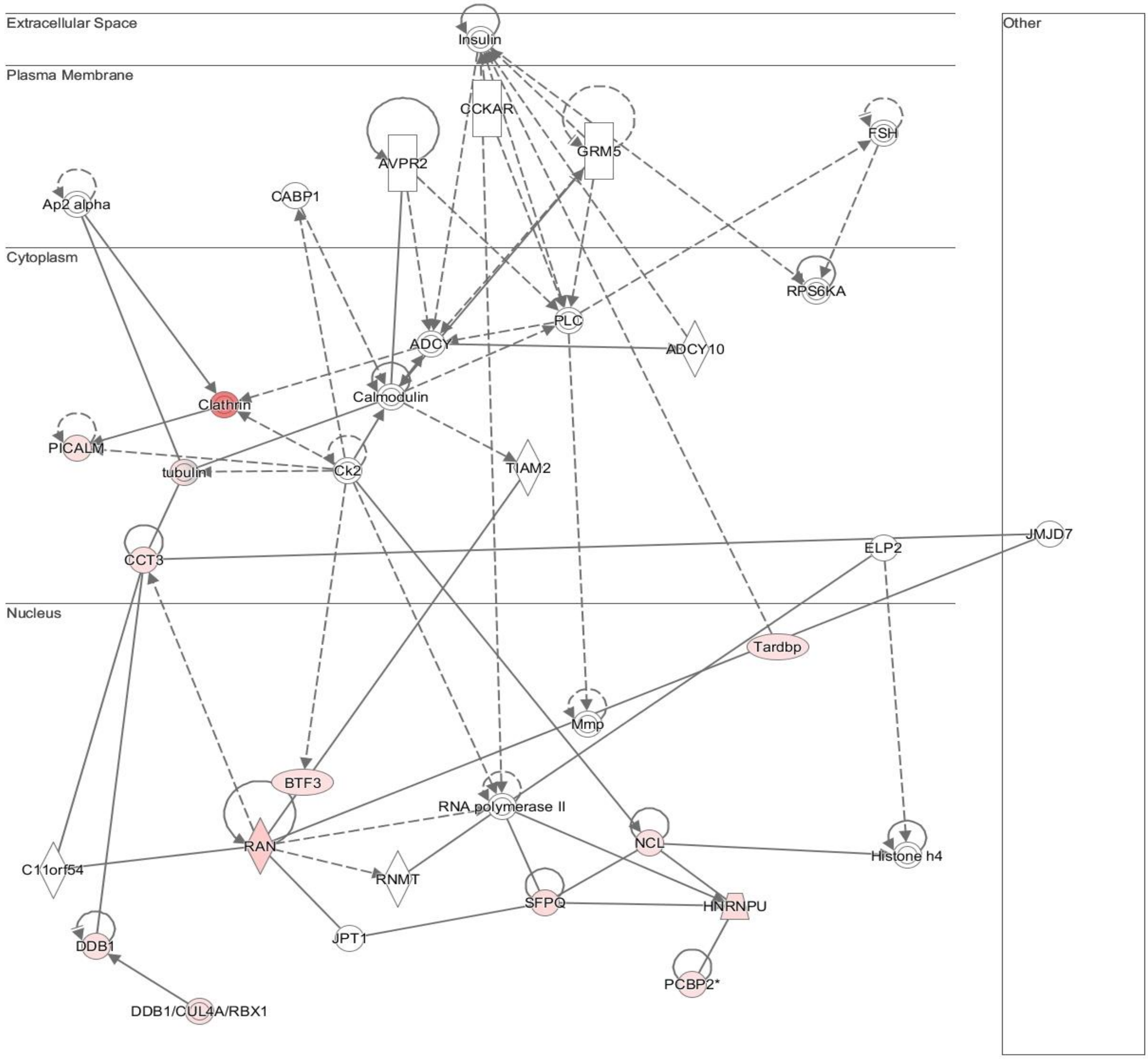

(**b**)

Canonical pathway	Target molecules	Z-score	P-value
Clathrin mediated endocytosis signaling	Ap2alpha, CK2, Clathrin, Insulin, PICALM	N/A	4.53E-04
Estrogen receptor signaling	ADCY10, ADCY, Mmp, PLC, RNA polymerase II	0.45	2.35E-03

Figure S9. IPA® network linked to development disorder, hereditary disorder and RNA post-transcriptional modification (**a**). The network was assembled from E_2-regulated proteins in the uterus of OVX rats through identification by label-free shotgun proteomics. Red symbols: upregulated, green symbols: downregulated by the hormone; solid line: direct relationship, dashed line: indirect relationship. See http://qiagen.force.com/KnowledgeBase/articles/Basic_Technical_Q_A/Legend for further details on symbols and relationships. (**b**) Top canonical pathways and their corresponding molecular targets associated with the network shown. Z-scores indicate activation or suppression of the corresponding pathway (positive or negative values, respectively; N/A: no prediction can be made).

(a)

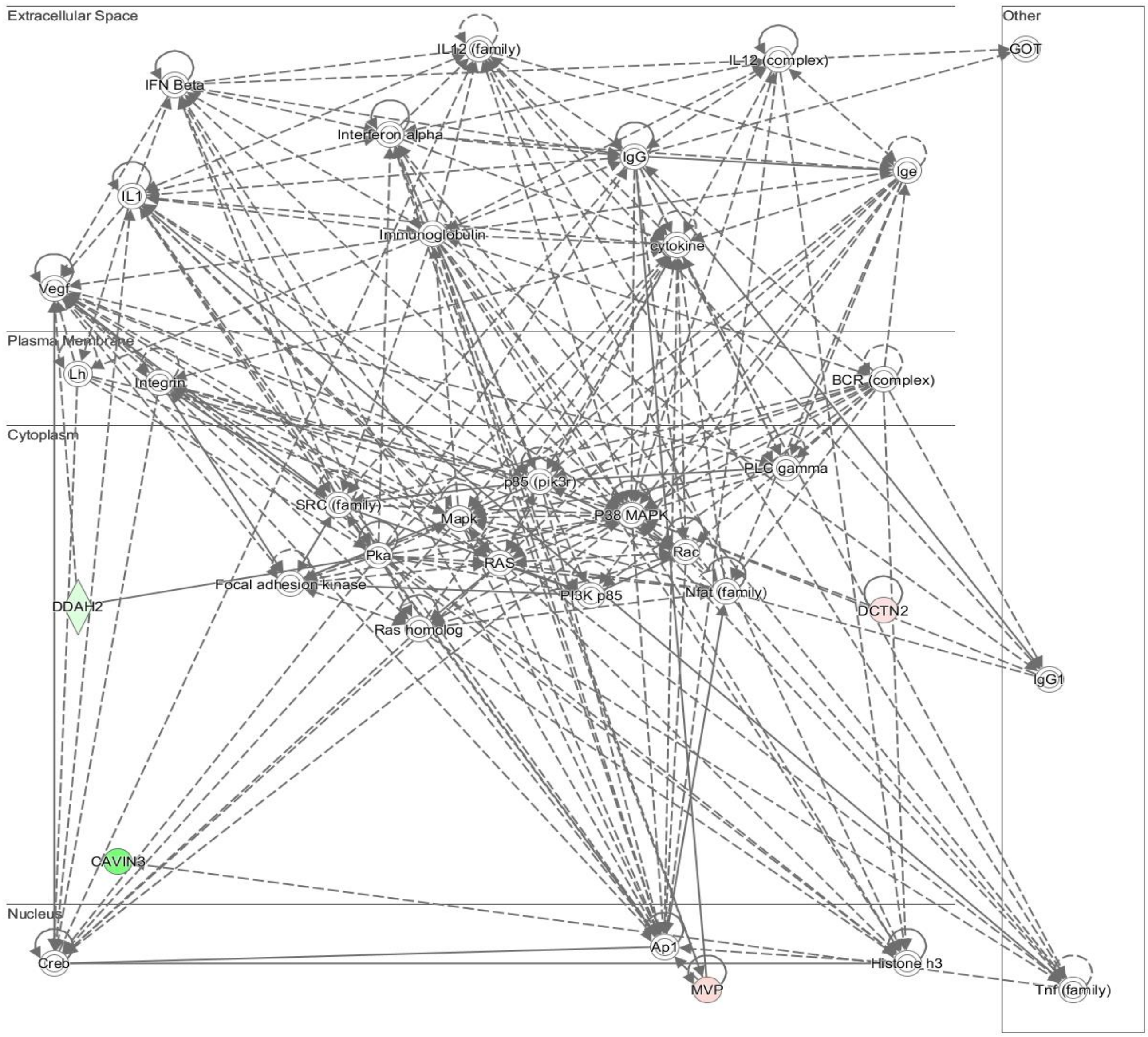

(**b**)

Canonical pathway	Target molecules	Z-score	P-value
Estrogen receptor signaling	Ap1, Creb, Mapk,p38 Mapk, p85, PI3K P85, Pka, PLC gamma, RAS, Ras homolog, SRC (Family), Vegf	0.45	2.35E-03
ILK signaling	Ap1, creb, focal adhesion kinase, integrin, mapk, p38 Mapk, p85, P13Kp85, Rac, Ras homolog, Tnf family, Vegf	1.4	6.32E-05

Figure S10. IPA® network linked to carbohydrate metabolism, drug metabolism and molecular transport (**a**). The network was assembled from E_2-regulated proteins in the uterus of OVX rats through identification by label-free shotgun proteomics. Red symbols: upregulated, green symbols: downregulated by the hormone; solid line: direct relationship, dashed line: indirect relationship. For further details on symbols and relationships, see http://qiagen.force.com/KnowledgeBase/articles/Basic_Technical_Q_A/Legend. Inset table (**b**) Top canonical pathways and their corresponding molecular targets associated with the network shown. Z-scores indicate activation or suppression of the corresponding pathway (positive or negative values, respectively; N/A: no prediction can be made).

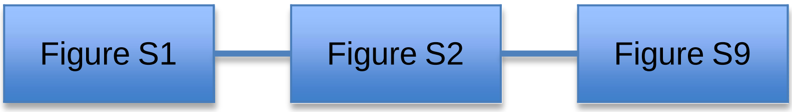

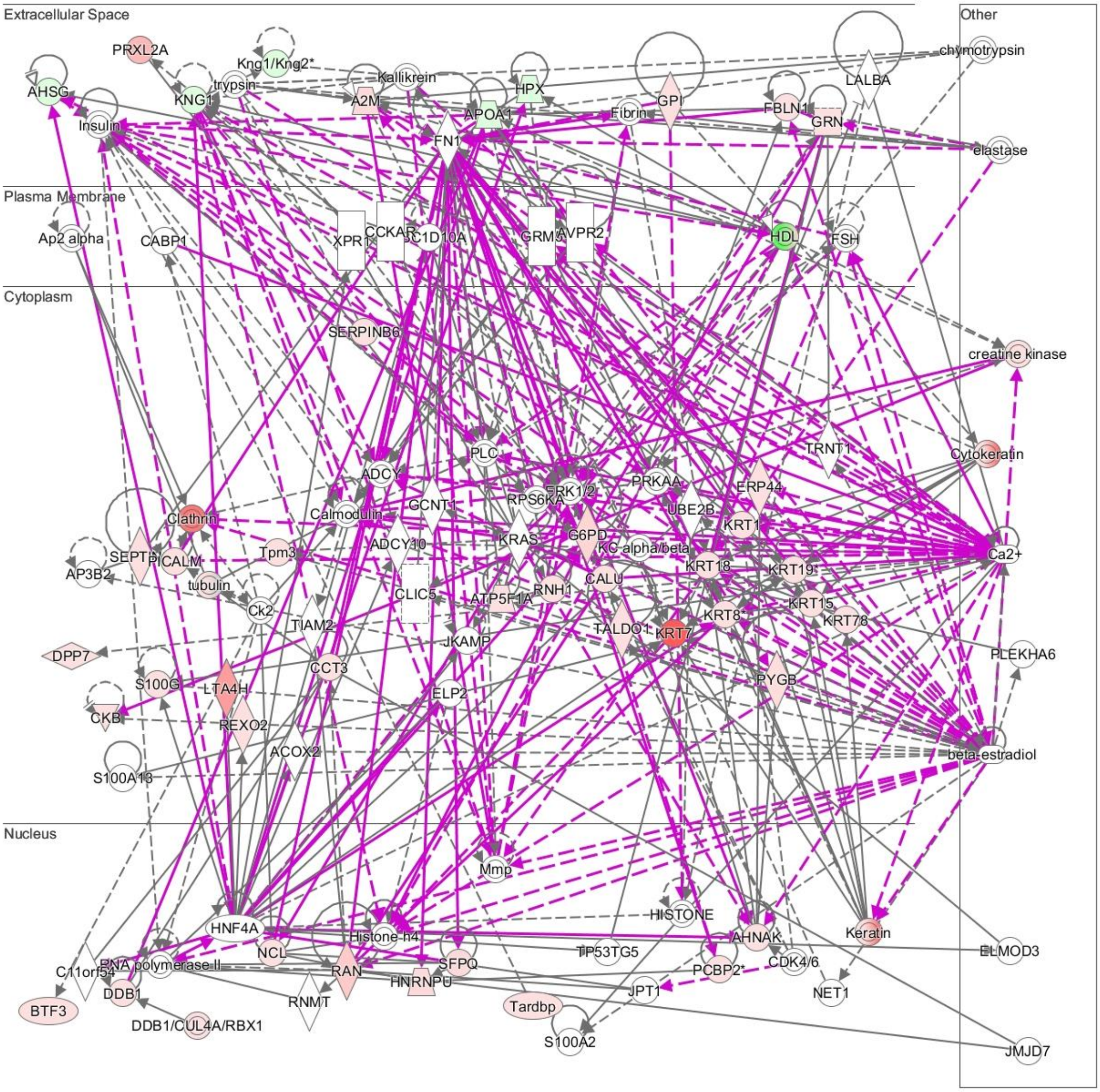

Figure S11. Overlapping IPA® networks.

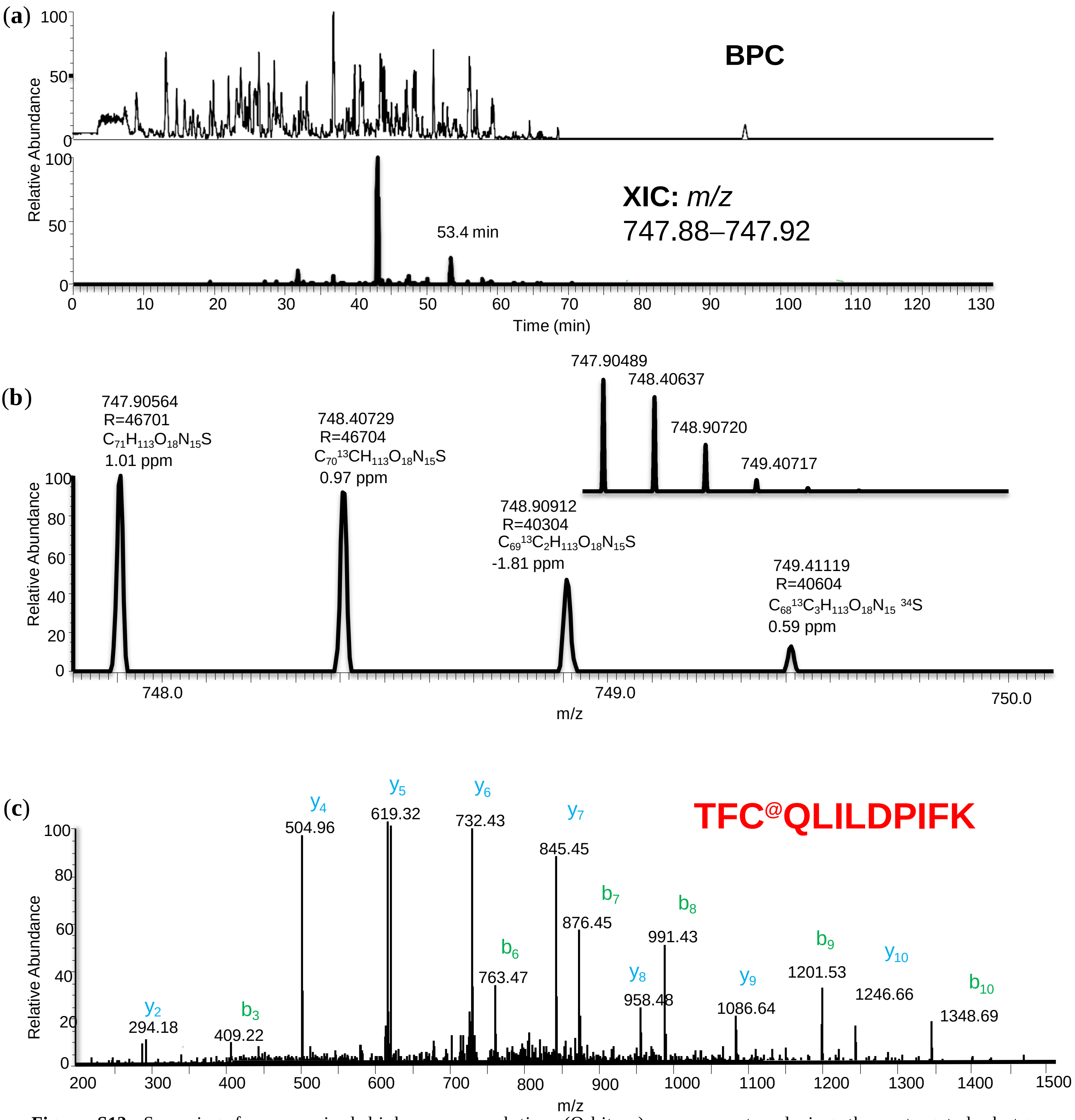

Figure S12. Screening from acquired high mass resolution (Orbitrap) mass spectra during the untargeted shotgun acquisitions for EEF2 as a prioritized uterine protein marker for targeted assay development. (**a**) Locating a proteotypic tryptic peptide of EEF2 by accurate mass of its doubly-charged ion $[M+2H]^{2+}$ (BPC: Base-peak ion chromatogram, XIC: Accurate-mass extracted ion chromatogram); (**b**) Verifying accurate masses and isotope peak distribution of the doubly-charged ions (R: mass resolution, inset: theoretical m/z and isotope peak distribution); (**c**) Confirming the peptide sequence TFC@QLILDPIFK by the acquired ion-trap MS/MS scan (b and y sequence ions marked according to nomenclature by Roepstorff and Fohlman [41]; C@ is carbamidomethylated cysteine).

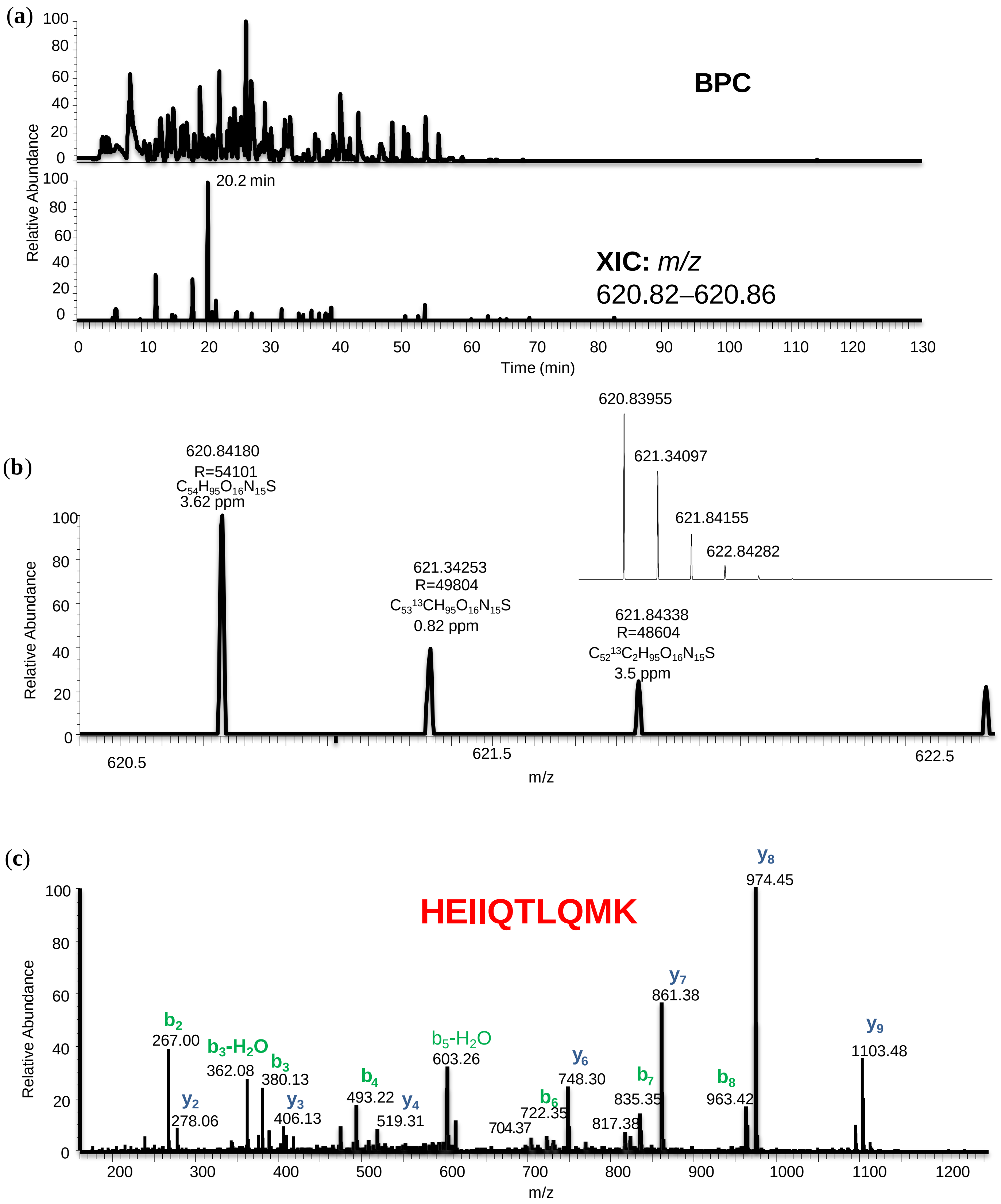

Figure S13. Screening from acquired high mass resolution (Orbitrap) mass spectra during the untargeted shotgun acquisitions for SELENBP1 as a prioritized uterine protein marker for targeted assay development. (**a**) Locating a proteotypic tryptic peptide of SELENBP1 by accurate mass of its doubly-charged ion $[M+2H]^{2+}$ (BPC: Base-peak ion chromatogram, XIC: Accurate-mass extracted ion chromatogram); (**b**) Verifying accurate masses and isotope peak distribution of the doubly-charged ions (R: mass resolution, inset: theoretical m/z and isotope peak distribution); (**c**) Confirming the peptide sequence HEIIQTLQMK by the acquired ion-trap MS/MS scan (b and y sequence ions marked according to nomenclature by Roepstorff and Fohlman [41]).

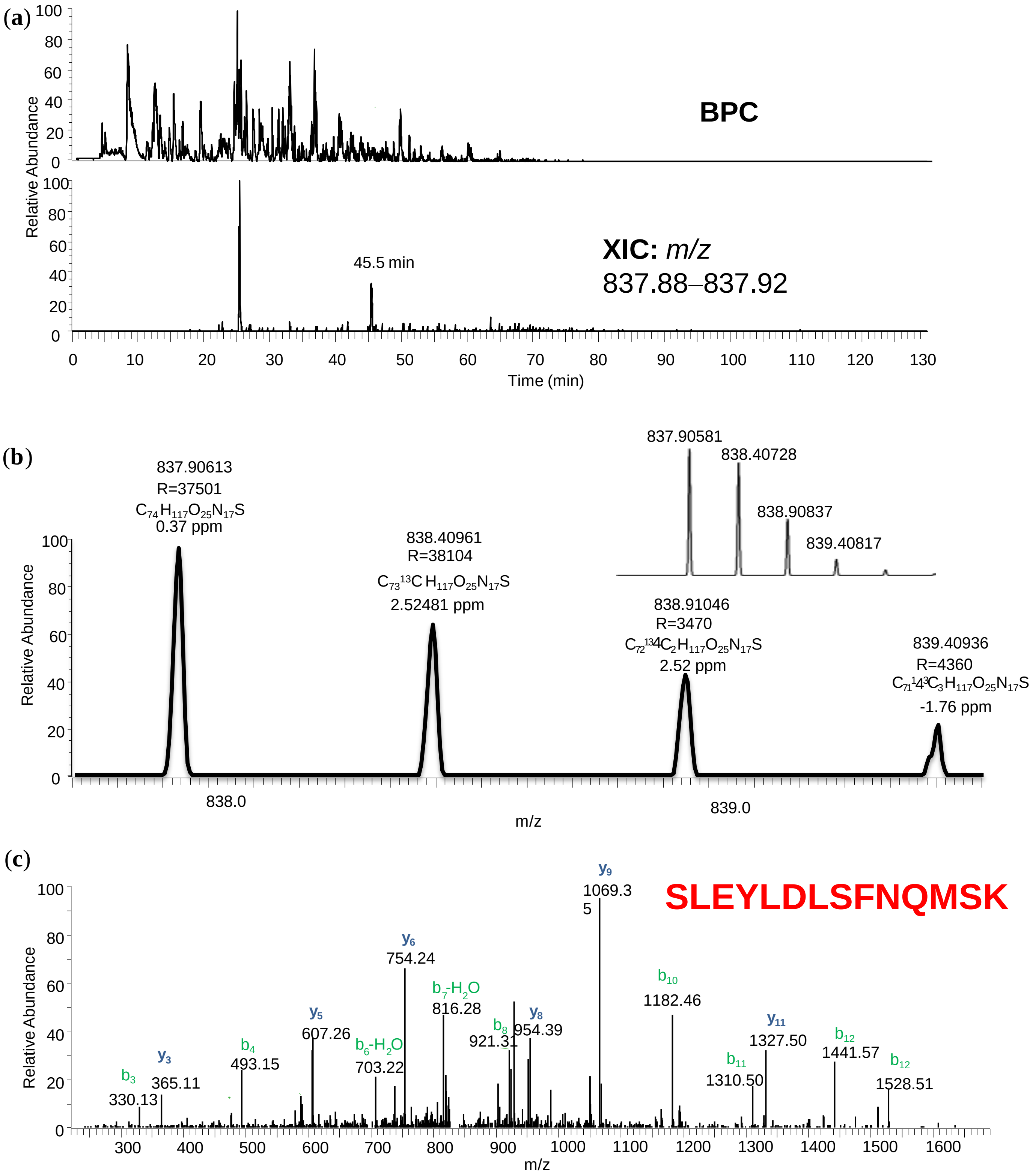

Figure S14. Screening from acquired high mass resolution (Orbitrap) mass spectra during the untargeted shotgun acquisitions for LUM as a prioritized uterine protein marker for targeted assay development. (**a**) Locating a proteotypic tryptic peptide of LUM by accurate mass of its doubly-charged ion $[M+2H]^{2+}$ (BPC: Base-peak ion chromatogram, XIC: Accurate-mass extracted ion chromatogram); (**b**) Verifying accurate masses and isotope peak distribution of the doubly-charged ions (R: mass resolution, inset: theoretical m/z and isotope peak distribution); (**c**) Confirming the peptide sequence SLEYLDLSFNQMSK by the acquired ion-trap MS/MS scan (b and y sequence ions marked according to nomenclature by Roepstorff and Fohlman [41]).
