# Supplementary material for: Proteomics Complementation of the Rat Uterotrophic Assay for Estrogenic Endocrine Disruptors: A Roadmap of Advancing High Resolution Mass Spectrometry-Based Shotgun Survey to Targeted Biomarker Quantifications
Source: Int J Mol Sci. 2021 Feb 8;22(4):1686. doi: 10.3390/ijms22041686 (PMC7914934; doi:10.3390/ijms22041686)
Supplement: Supplementary file 1 [file ijms-22-01686-s001.zip › ijms-1078409_TableS3.pdf]

Table S3. List of optimized MRM transitions used for relative quantitation analysis of TGM2, EEF2, SELENBP1 and LUM with isotope-labeled internal standards.

Peptide Sequence	NAT Precursor *m/z*	NAT Fragment *m/z*	SIS Precursor *m/z*	SIS Fragment *m/z*	Collison Energy	Fragment Ion	Retention Time Segment (in min)	Dwell Time (in ms)	Protein ID
YSGC@LTESNLIK	692.8	804.5	696.9	812.7	24	**y7**	0-12	17	TGM2
YSGC@LTESNLIK	692.8	917.6	696.9	925.8	24	y8	0-12	17	TGM2
SEGTYC@C@GPVSVR	736.3	934.7	741.4	944.6	33	y8	0-12	17	TGM2
SEGTYC@C@GPVSVR	736.3	614.5	741.4	624.3	24	**y6**	0-12	17	TGM2
EGIPALDNFLDKL	722.8	573.5	726.4	576.8	23	**y10^{++}**	15-45	25	EEF2
EGIPALDNFLDKL	722.8	186.8	726.4	186.8	29	b2	15-45	25	EEF2
TFC@QLILDPIFK	747.8	504.2	752	512.3	29	**y4**	15-45	25	EEF2
TFC@QLILDPIFK	747.8	845.9	752	853.6	25	y7	15-45	25	EEF2
LILPSIISSR	549.9	759.5	554.9	769.5	24	y7	12-15	25	SELENBP1
LILPSIISSR	549.9	227.0	554.9	227.0	20	**b2**	12-15	25	SELENBP1
HEIIQTLQMK	620.9	266.9	624.9	266.9	28	**b2**	0-12	17	SELENBP1
HEIIQTLQMK	620.9	362.0	624.9	362.0	33	b3-H2O	0-12	17	SELENBP1
NNQIDHIDEK	613.3	257.9	617.3	265.9	24	**y2-H_2O**	0-12	17	LUM
NNQIDHIDEK	613.3	275.9	617.3	283.9	27	y2	0-12	17	LUM
SLEYLDLSFNQMSK	838.1	841.4	842.1	849.5	25	**y7**	12-15	25	LUM
SLEYLDLSFNQMSK	838.1	1069.6	842.1	1077.8	23	y9	12-15	25	LUM

NAT is endogenous proteotypic tryptic peptide.
SIS is stable isotope labeled peptide labeled on C-terminal amino acid, giving mass shift of +8 Da (from $^{13}C_6{}^{15}N_2$-lysine), +10 Da (from $^{13}C_6{}^{15}N_4$-arginine), and +7 Da (from $^{13}C_6{}^{15}N$-leucine) compared to non-labeled counterpart (NAT).
C@ is carbamidomethylated cysteine.
Bolded fragment ion indicates quantifier ion, and the other fragment ion for each peptide is qualifier ion.
